# Supplementary material for: Molecular Changes in Dengue Envelope Protein Domain III upon Interaction with Glycosaminoglycans
Source: Pathogens. 2020 Nov 11;9(11):935. doi: 10.3390/pathogens9110935 (PMC7697694; doi:10.3390/pathogens9110935)
Supplement: Supplementary file 1 [file pathogens-09-00935-s001.pdf]

Supplementary materials

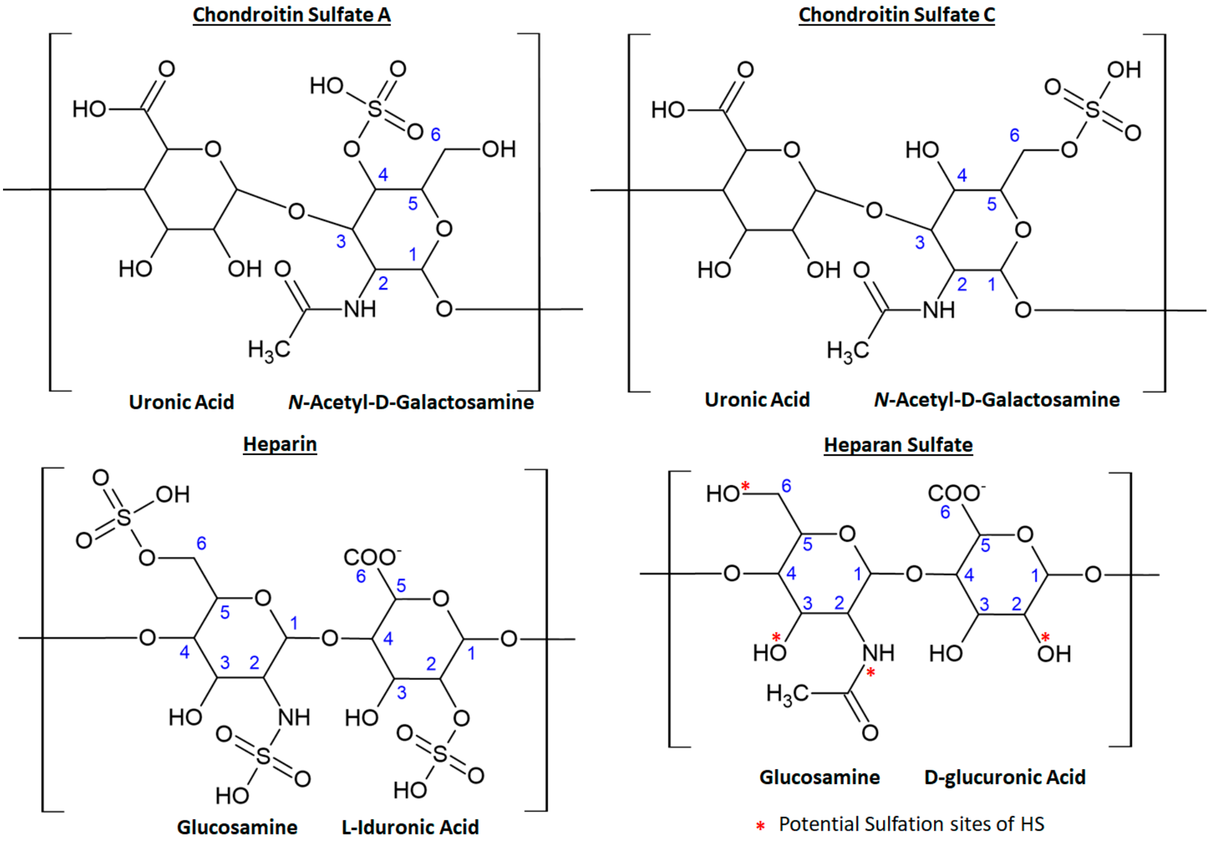

Figure S1. Glycosaminoglycans are distinguished by their repeating disaccharide subunits, sulfation state, and linker positioning. Chondroitin Sulphate A and C differ in the positioning of their sulfate groups; CSA is predominantly sulfated at the 4' position of its N-acetyl-D-Galactosamine saccharide, whilst CSC is sulfated predominantly at the 6' position. The level of sulfation at both groups may vary dependent upon the species origin of the GAG sample. Heparin has three potential sulfation positions per disaccharide subunit, of which an average of 2.7 are sulfated. Heparan sulfate is a lesser sulfated derivative of heparin, and in contrast has four potential sulfation positions of which an average of only one of these is sulfated per disaccharide subunit. Furthermore, the chirality of the C_5 carboxylic acid group gives rise to either a D-glucuronic or L-iduronic epimer as observed in heparan sulfate or heparin, respectively.

10<br>MRGSHHHHHH	20<br>GMASMTGGQQ	30<br>MGRDLTDDDD	40<br>KTRTGSQENL	50<br>YFQG/SGMSYS	60<br>MCTGKFKIVK
70<br>EIAETQHGTI	80<br>VIRVQYEGDG	90<br>SPCKIPFEIT	100<br>DLEKRHVLGR	110<br>LITVNPIVTE	120<br>KDSPVNIEAE
130<br>PPFGDSYIII	140<br>GVEPGQLKLN	WFKK			

Figure S2. Full amino acid sequence for the EDIII construct used in this study. Our construct closely resembles the strain Jamaica/1409/1983, (amino acids 296-394) with one residue differing (M90T, shown in pink). His-tag shown in red, TEV cleavage site in green, and EDIII in blue.

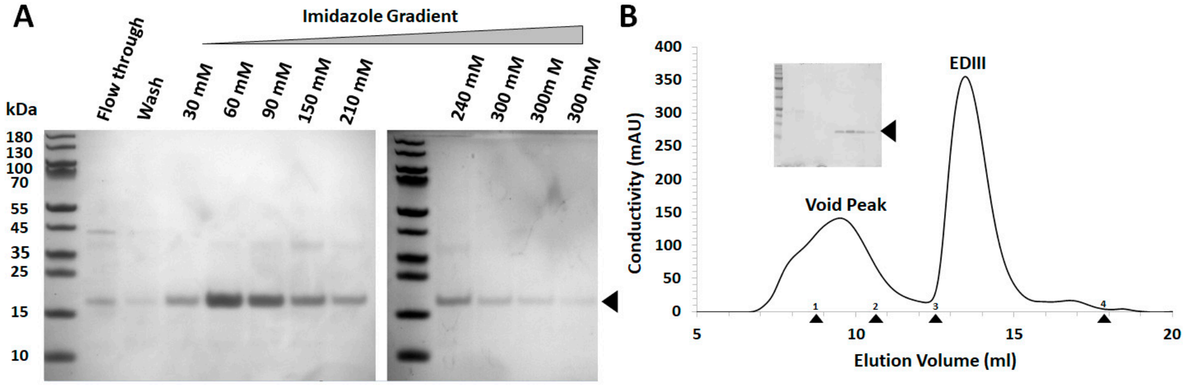

Figure S3. Purification of recombinant EDIII. (A) IMAC purification of refolded, un-cleaved EDIII, showing flow through (FT), wash (W), elution fractions of increasing imidazole concentration. EDIII is indicated by an arrow at an approximate size of 16kDa. (B) Size exclusion chromatography, showing major peak for TEV-cleaved EDIII at an apparent molecular weight of 11.4kDa (elution volume - 13.47ml). Molecular weight markers along X-axis corresponding to elution volumes of 1 - γ-globulin, 2 - Ovalbumin, 3 - Myoglobin, and 4 - Vitamin B12. SDS-PAGE analysis of peak fractions as inset; molecular weight ladder as shown in supplementary figure 1A.

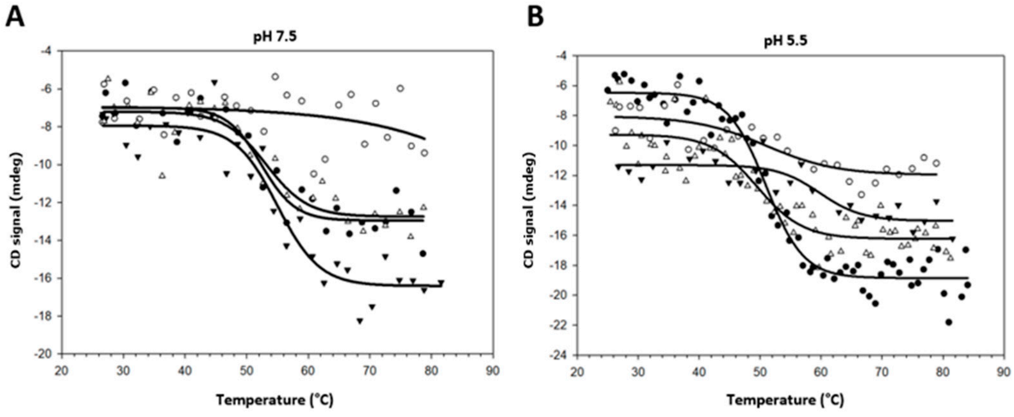

Figure S4. Sigmoidal fit of CD wavelength/temperature interval data conducted at 216nm. EDIII alone (black circles) and with CSCi (empty triangles), H (empty circles), and CSCii (black triangles) at pH 7.5 (A) and pH 5.5 (B). Data for EDIII with heparin at pH 7.5 could not be fit.

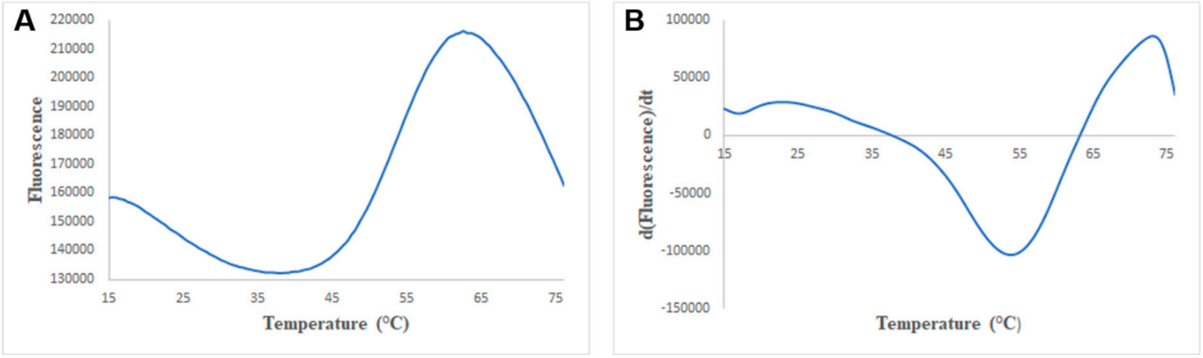

Figure S5. Differential scanning fluorimetry for His-tagged EDIII. (A) normalised fluorescence data (B) derivative plot of fluorescence data. EDIII was measured every 0.5 °C, data averaged from four independent repeats. A Tm of 52.3°C was calculated from the minimum of the derivative plot.

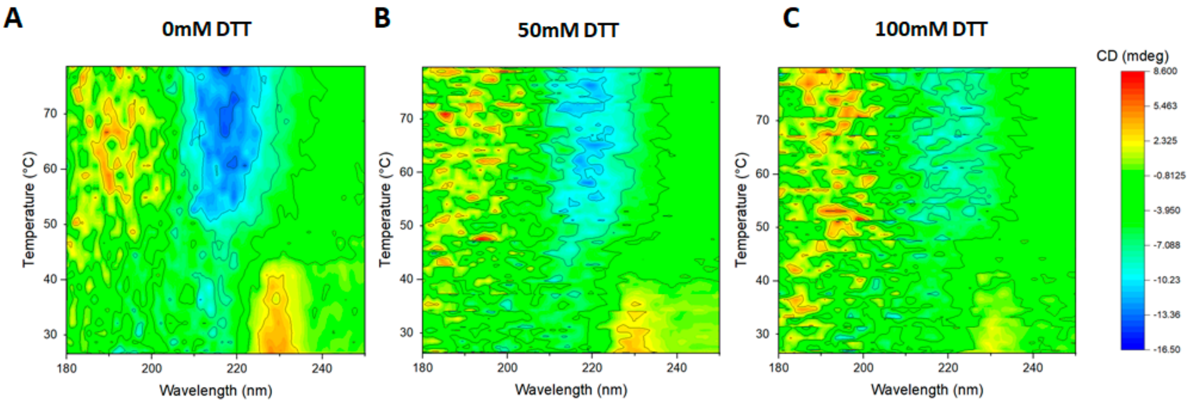

Figure S6. Increasing concentrations of DTT cause the maximum at 225nm to disappear at lower temperatures. EDIII was incubated with increasing concentrations of DTT - 0mM (A), 50mM (B), and 100mM (C), overnight prior to analysis by wavelength/temperature interval CD analysis. Figure created using Origin software.

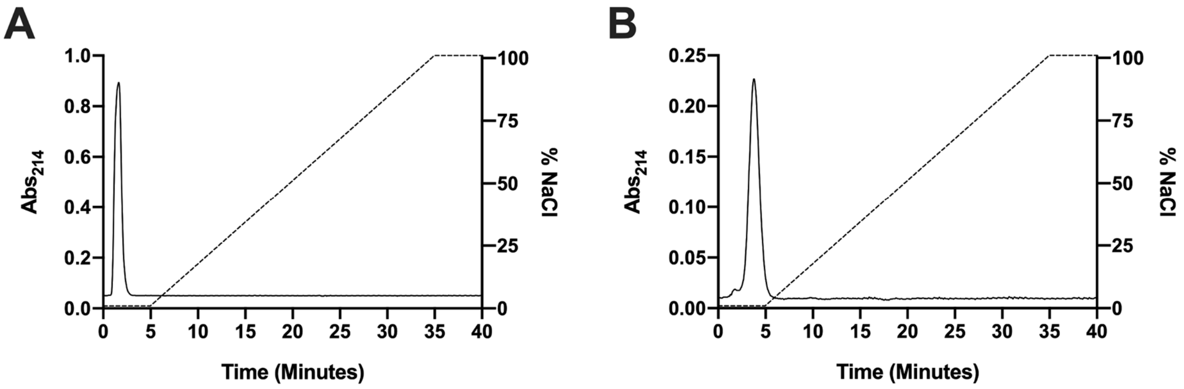

Figure S7. Heparin affinity chromatography with EDIII. Protein was run on a heparin column at (A) pH 5.5 and (B) 7.5. EDIII did not appear to bind to the column and was eluted before the salt gradient started (dotted line).

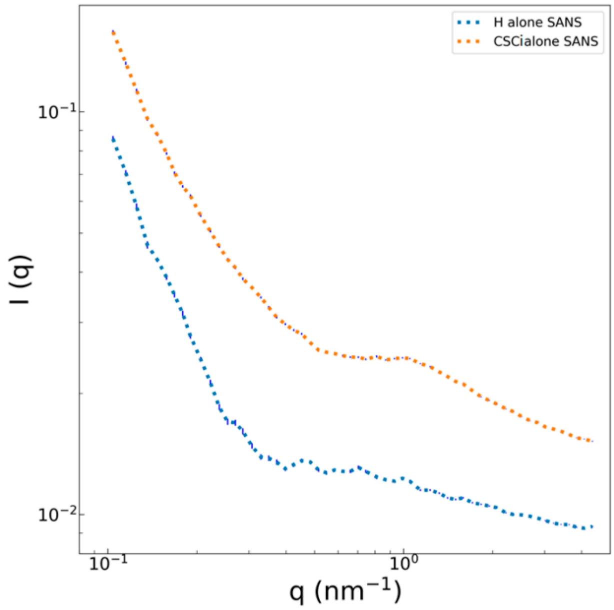

Figure S8. SANS scattering data of Heparin (blue) and CSCi (orange). 30 mg/ml of GAGs was measured in D_2O. An increase in scattering can be observed at low q.

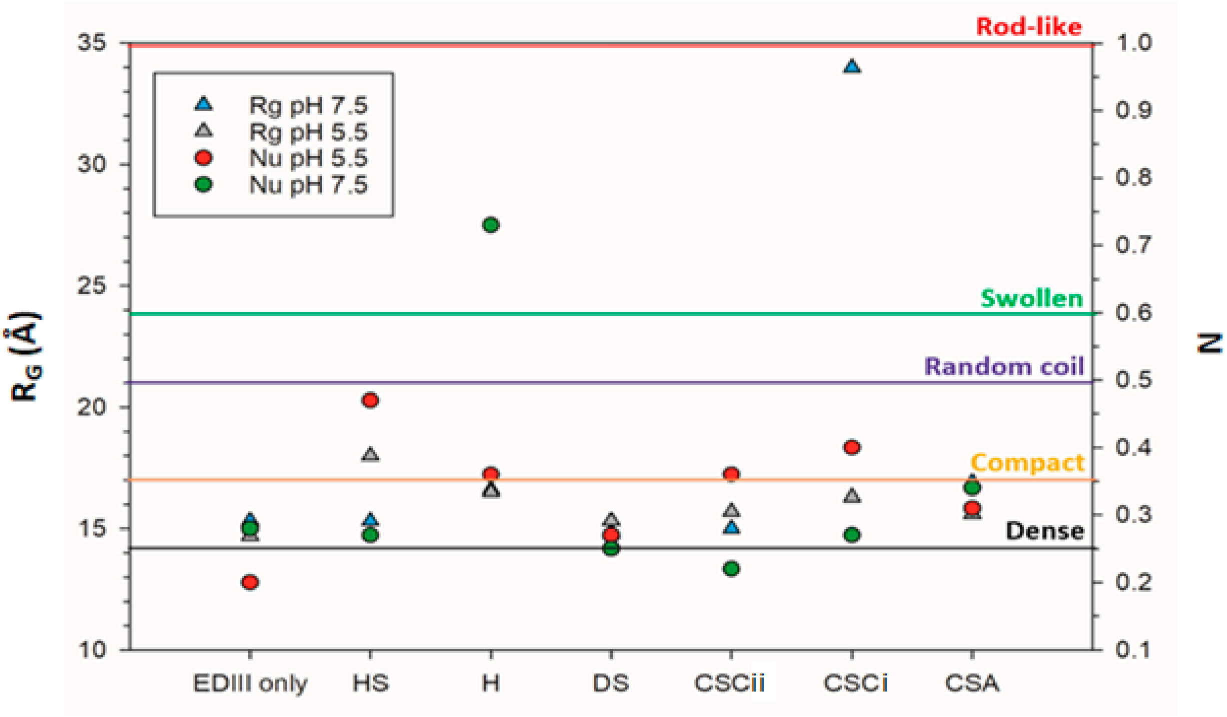

Figure S9. Guinier and Nu analysis of EDIII alone and with GAGs from SANS data. Guinier (triangles) and Nu (circles) analysis was conducted at pH 7.5 and 5.5. Comparative levels of compactness are shown as lines across the graph, with increasing levels of disorder represented by Nu (N).

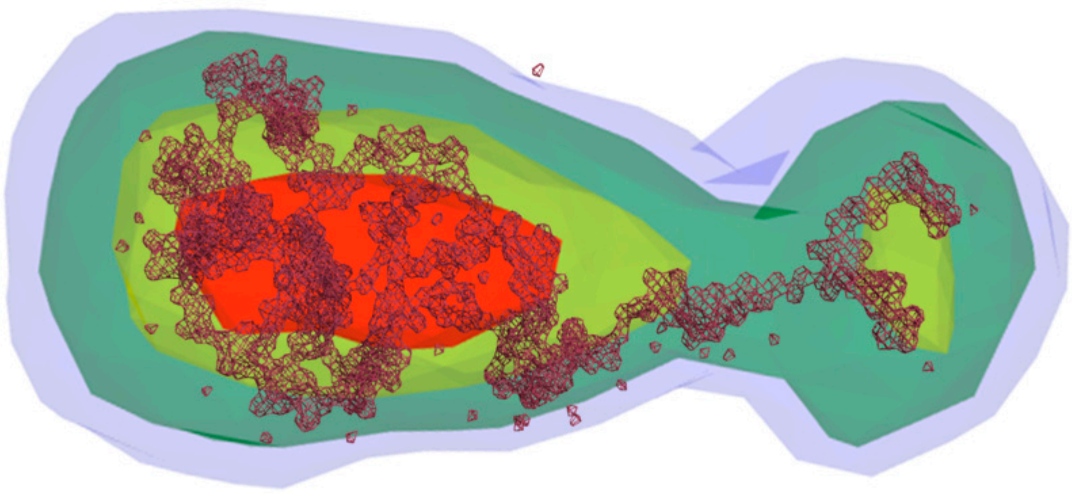

Figure S10. GASBOR and DENSS models of EDIII with CSC. GASBOR mass distribution represented in red mesh, and DENSS in blue, green, light green, and red (with increasing density). Both models show an uneven mass distribution with mass concentrated more towards one end.

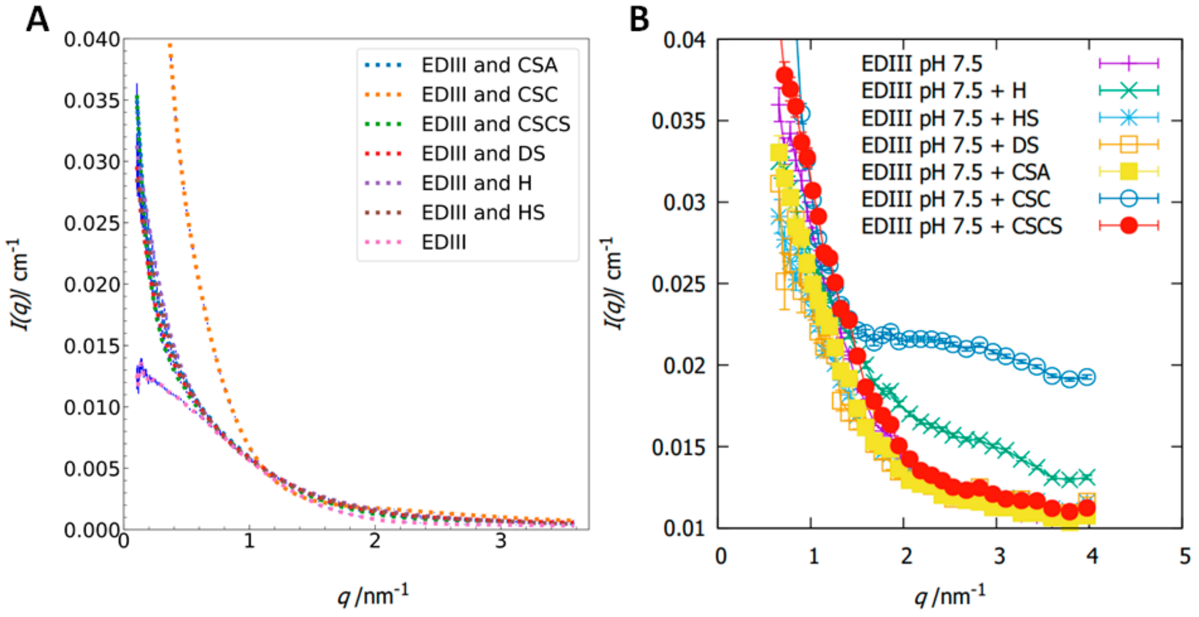

Figure S11. Linear scale SAXS and SANS spectra of EDIII alone and with different GAGs at pH 7.5. (A) SAXS spectra show no change between EDIII only (pink) and ant samples incubated with GAGs. (B) SANS spectra show changes at the molecular level when EDIII was incubated with both H (green) and CSCi (blue), as evidenced by a slower intensity decay (lower Porod exponent) in the high-q regions.

Supplementary Table 1. NRMSD values for fitting of CD wavelength spectra in secondary deconvolution fitting via BeStSel.

Sample	pH	NRMSD
	4	0.13067
EDIII - untagged	5.5	0.11086
	7.5	0.11746
EDIII		0.10005
EDIII with H		0.08928
EDIII with DS	5.5	0.11706
EDIII with CSC		0.08439
EDIII with CSA		0.13557
EDIII		0.11356
EDIII with H		0.0721
EDIII with DS	7.5	0.13502
EDIII with CSC		0.09257
EDIII with CSA		0.05388
